# Body mass index and thoracic subcutaneous adipose tissue depth: possible implications for adequacy of chest compressions

**DOI:** 10.1186/s13104-017-2918-9

**Published:** 2017-11-07

**Authors:** Paul Secombe, Ross Sutherland, Richard Johnson

**Affiliations:** 1 School of Medicine, Flinders University, Bedford Park, South Australia Australia; 2 Intensive Care Consultant, Intensive Care Department, Alice Springs Hospital, Gap Road Alice Springs, Alice Springs, Northern Territory Australia; 3 Department of Emergency Medicine, Flinders Medical Centtre, Adelaide, South Australia Australia; 4 Emergency and Retrieval Medicine Consultant, Retrieval Medicine, Alice Springs Hospital, Gap Road Alice Springs, Alice Springs, Northern Territory Australia; 5 Honorary Research Fellow, Baker Institute, Alice Springs Hospital, Gap Road Alice Springs, Alice Springs, Northern Territory Australia

**Keywords:** Body mass index, Cardiopulmonary resuscitation, Obesity, Subcutaneous fat, Tomography

## Abstract

**Objective:**

Adequacy of cardiopulmonary resuscitation relies on compression of the thoracic cage to produce changes in intra-thoracic pressures sufficient to generate a pressure gradient. In order to evaluate the efficacy of cardiopulmonary resuscitation in morbid obesity, it is first necessary to determine the depth of thoracic subcutaneous adipose tissue (SAT) and to correlate this with body mass index (BMI).

**Results:**

Computerised-tomography images of the thorax of 55 patients with a diagnosis of obesity or morbid obesity (mean BMI 45.95 kg/m^2^) were evaluated to determine the depth of SAT at the level at which chest compressions would be applied by a trained rescuer, and correlated with BMI. Mean anterior SAT was 36.53 mm, and mean posterior SAT was 50.73 mm. There was a significant correlation between BMI and anterior and posterior SAT for males (p < 0.05 for both), and females (p < 0.05 for both). The slope of the functions was considered sufficiently close to allow combining the data. This also showed a significant correlation between SAT and BMI (p < 0.01 for both). Both anterior and posterior SAT is correlated with BMI. This data allows development of a model to explore the efficacy of chest compressions in morbid obesity.

**Electronic supplementary material:**

The online version of this article (10.1186/s13104-017-2918-9) contains supplementary material, which is available to authorized users.

## Introduction

International epidemiological studies demonstrate that the prevalence of obesity (body mass index (BMI) ≥ 30 kg/m^2^) has risen over the past three decades and is increasing [1–3]. Obesity is associated with a range of co-morbidities that may predispose to cardiac arrest including ischaemic heart disease, hypertension, and uni- or bi-ventricular dysfunction or failure [4–6].

Adequate cardiopulmonary resuscitation (CPR) forms the cornerstone of both basic and advanced life support [7–9]. Inadequate CPR is associated with poor clinical outcomes, and, after delay in treatment, is the strongest predictor of good outcomes [8, 10, 11].

The primary aim of CPR is to “temporarily maintain circulation sufficient to preserve brain function” [12]. The underlying physiologic principles are only partially understood and remain controversial [11]. In brief, however, the positive intra-thoracic pressure generated by compression of the thorax produces a positive pressure gradient in the aorta, maintaining blood flow to crucial organs—the goal being maintenance of cardiac and cerebral perfusion [11]. The chest decompression phase produces negative intra-thoracic pressure drawing blood into the thoracic space allowing passive refilling of the right sided cardiac chambers.

Failing to allow time for the cardiac chambers to refill through either incomplete recoil, or undertaking compressions at a rate that is too fast reduces perfusion pressures [11, 13, 14]. For compressions to be adequate the European Resuscitation Council (ERC) recommend compressions be undertaken at a rate of 100–120 beats/min to a depth of one-third of the depth of the chest (which equates to approximately 5 cm in adults) allowing complete recoil of the chest after each compression [15].

What appears missing in these observations is that compression and decompression of the thoracic cage is necessary to achieve these results, not the chest itself. While this appears to overly emphasise semantics, it may be important in the setting of morbid obesity when the thoracic cage is encased in a layer of adipose tissue.

As part of a larger study investigating the adequacy of trained rescuer chest compressions in morbid obesity, we sought to measure the depth of subcutaneous adipose tissue (SAT) through which compressions must pass in order to impact the thoracic cage and correlate depth of SAT with BMI.

## Main text

### Methods

**Fig. 1:**
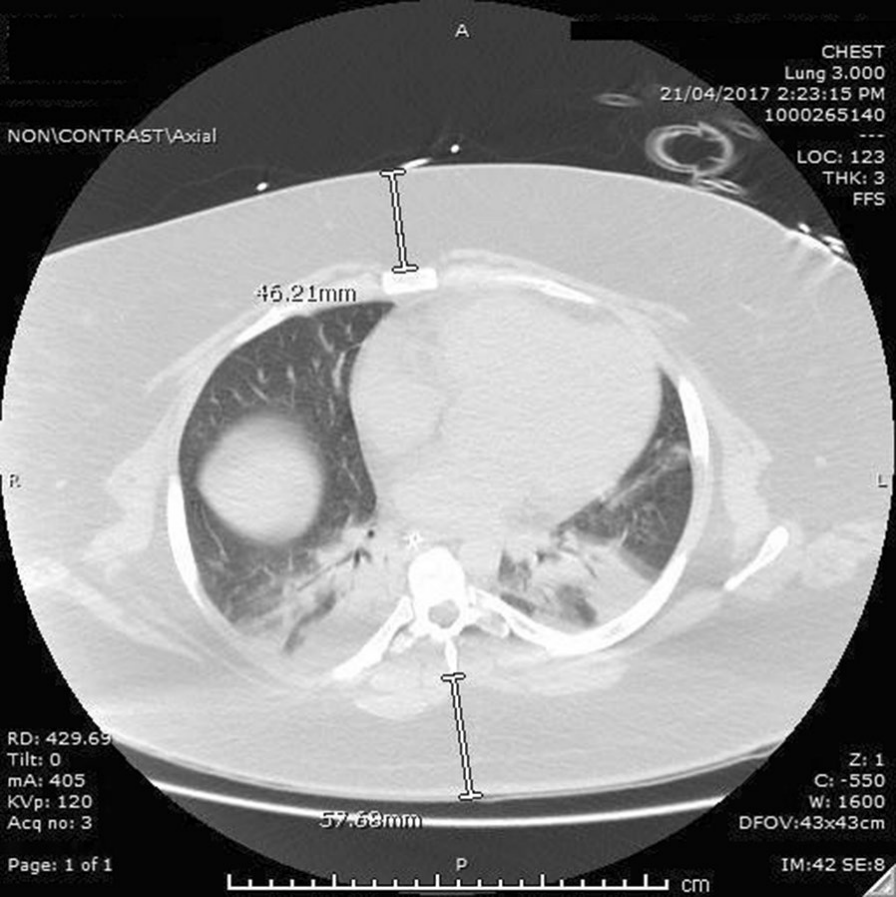
Example CT thorax image demonstrating sites at which measurement of SAT were made in the anterior–posterior plane

Retrospective radiological review undertaken at the Alice Springs Hospital (ASH), a 186 bed regional hospital in Central Australia. Patients discharged from the ASH between 2011 and 2016 with a diagnosis of “obesity” or “morbid obesity” were identified using the Northern Territory Government electronic discharge summary system. The subgroup of these patients who had undergone both computerised tomography (CT) of the thorax and had a weight (allowing calculation of BMI) or BMI recorded within 6 months of the scan had images extracted for analysis. Measurements of SAT were made in the anterior–posterior plane at the ‘ideal’ site of chest compressions, this is to say between the skin surface and the sternum at the 4th costal cartilage anteriorly and the skin surface and the tip of the 3rd spinous process posteriorly (Fig. 1) [16]. These sites were chosen as they represent the tissues which are likely to re-distribute forces before movement of the thoracic cage occurs when rescuers perform compressions at the recommended location for CPR [15].

#### Ethics approval and consent to participate

Ethics approval, including waiver of consent on the basis that subjects had no direct involvement and that data was collected retrospectively from pre-existing data repositories, was obtained from the Central Australian Human Research Ethics Committee (HREC-16-445).

#### Statistical analysis

Data was analysed in Statistica™ (StatSoft Incorporated, Tusla, Oklohoma; http://www.statsoft.com). Data is reported as mean (standard deviation) for normally distributed data or number (percentage) for categorical variables. Data was assessed for normality using the Shapiro–Wilks normality test. The Pearson product-moment correlation coefficient was used to measure the linear correlation between SAT and BMI. No adjustment was made for multiple comparisons and results were considered significant at p < 0.05.

To identify a correlation coefficient of 0.4 with α = 0.05 and β = 0.8, a sample size of 46 would be required. To allow for some imaging that would be inappropriate and/or fail to demonstrate posterior or anterior SAT, a total sample of 50 was aimed for.

### Results

A search of the NTG electronic discharge system identified 195 unique patients with a discharge diagnosis of obesity or morbid obesity. Of these 55 (22 males and 33 females) had a CT thorax and a weight or BMI recorded within 6 months of the imaging (Additional file 1). Demographic data for these patients is provided in Table 1. Only 51 measurements of posterior SAT were possible due to clipping of the posterior margins of the image making identification of the skin surface impossible. Shapiro–Wilks was non-significant for dependent and independent variables.

**Table 1:** Demographic data for obese patients with CT thorax images

	Males (n = 22)	Females (n = 33)	All (n = 55)
Age at time of CT (years)	51.91 (10.78)	49.42 (12.86)	50.41 (12.03)
Indigenous Australian (n)	9 (41%)	24 (73%)	33 (60%)
Mean weight (kg) (n = 53)	143.09 (20.01) (n = 21)	121.77 (16.78) (n = 32)	130.22 (20.80)
Mean height (cm) (n = 53)	175.13 (9.87) (n = 21)	162.31 (6.88) (n = 32)	167.72 (10.25)
Mean BMI (kg/m^2^)	45.39 (6.07)	46.32 (6.36)	45.95 (6.21)
Mean anterior SAT (mm)	36.75 (9.61)	36.38 (7.3)	36.53 (8.21)
Mean posterior SAT (mm) (n = 51)	48.47 (15.03) (n = 22)	54.21 (10.69) (n = 29)	50.73 (13.18)

Data is presented as absolute number (and percentage), mean (and SD) or median (and IQR)

**Fig. 2:**
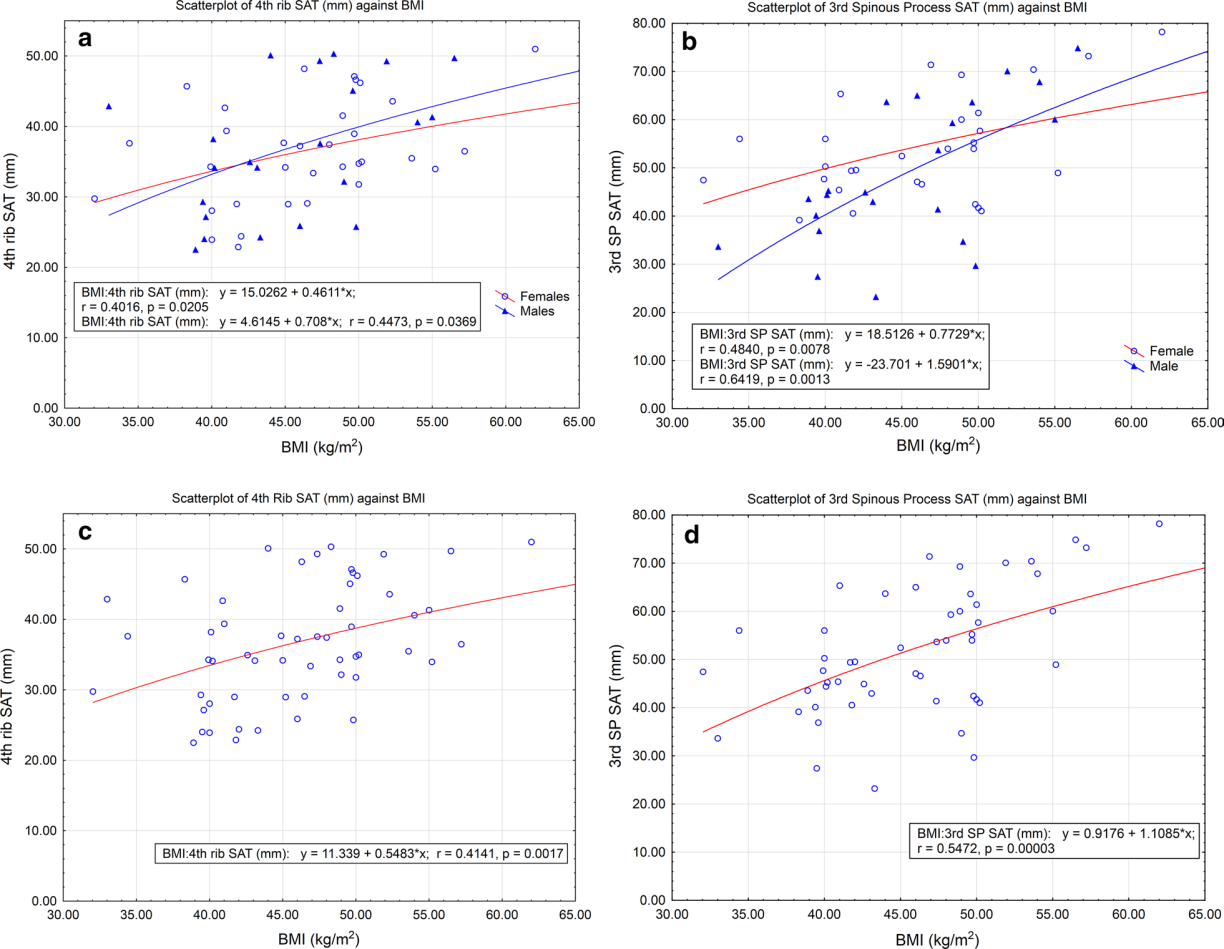
Scatterplot of SAT comparing males and females (**a** anterior SAT, **b** posterior SAT) and combined data for anterior SAT (**c**) and posterior SAT (**d**)

There was a significant correlation between BMI and both anterior and posterior SAT for males (r = 0.45, p = 0.04; r = 0.64, p < 0.01 for anterior and posterior respectively) and females (r = 0.40, p = 0.02; r = 0.48, p < 0.01, Fig. 2, a and b). The slope of the correlation was considered sufficiently close to allow combining male and female data (r = 0.41, p < 0.01; r = 0.55, p < 0.01 for anterior and posterior SAT respectively, Fig. 2, c and d).

### Discussion

When applying CPR to morbidly obese patients, forces transmitted to the thoracic cage must first pass through the subcutaneous adipose tissues. It is plausible that much of the force generated at the skin surface is displaced through the anterior and posterior subcutaneous adipose tissues (both between skin surface and the sternum, and between the spine and the skin surface). In order to better explore the implications of chest compressions in morbid obesity it is first necessary to construct a model that predicts SAT in obesity.

A search of the literature failed to identify any studies that have specifically explored the depth of SAT which may disperse these forces in the morbidly obese population, necessitating this observational study. A study by Lee and colleagues examined the relationship between internal and external anterio-posterior thoracic diameter. Although their major finding was a statistically significant relationship between these parameters and BMI, only 6% of the cohort had a BMI of > 30 kg/m^2^. This therefore represents a different population to that in this study and does not provide the data with which to guide the construction of a physical model to explore the adequacy of compressions in the morbidly obese patient [17].

This study demonstrates that thoracic SAT can be predicted from BMI with a reasonable degree of accuracy. Strengths of this observational study include the physiological intuitiveness of the underlying assumption, the relatively large sample size, and the use of pre-existing radiological images that have been taken while patients are in the same position as they would be during CPR.

## Limitations

There are, however, several limitations to our study. Firstly, there are a significant proportion of Australian Indigenous patients in this cohort. Indigenous Australians have a predisposition to the metabolic syndrome and central adiposity, which may skew these results [18–22]. Secondly, it was not possible to measure lateral SAT due to clipping of the lateral margins of the image in an effort to reduce the radiation dose received by patients. A prospective study using alternative imaging (such as ultrasound) may be warranted to explore this further. Thirdly, computerised tomography was used to determine the depth of SAT. While this seems intuitively reasonable there is no empirical evidence to support this, and alternative imaging modalities (such as ultrasound), may be better. Finally, we identified patients using a discharge diagnosis of obesity or morbid obesity. This necessarily creates a biased sample with a right skew to the data. Further prospective population studies would mitigate this risk.

In conclusion, this data demonstrates a clear and significant relationship between BMI and both anterior and posterior SAT. This should allow construction of a model to explore the efficacy and adequacy of chest compressions on a morbidly obese mannequin.

## Additional file

**Additional file 1.** Flow of patients through study. CONSORT type flow diagram.

## Abbreviations

BMI: body mass index
CPR: cardiopulmonary resuscitation
ERC: European Resuscitation Council
SAT: subcutaneous adipose tissue
ASH: Alice Springs Hospital
CT: computerised tomography
