# Supplementary figures and images for: Body mass index and thoracic subcutaneous adipose tissue depth: possible implications for adequacy of chest compressions
Source: BMC Res Notes. 2017 Nov 7;10:575. doi: 10.1186/s13104-017-2918-9 (PMC5678571; doi:10.1186/s13104-017-2918-9)

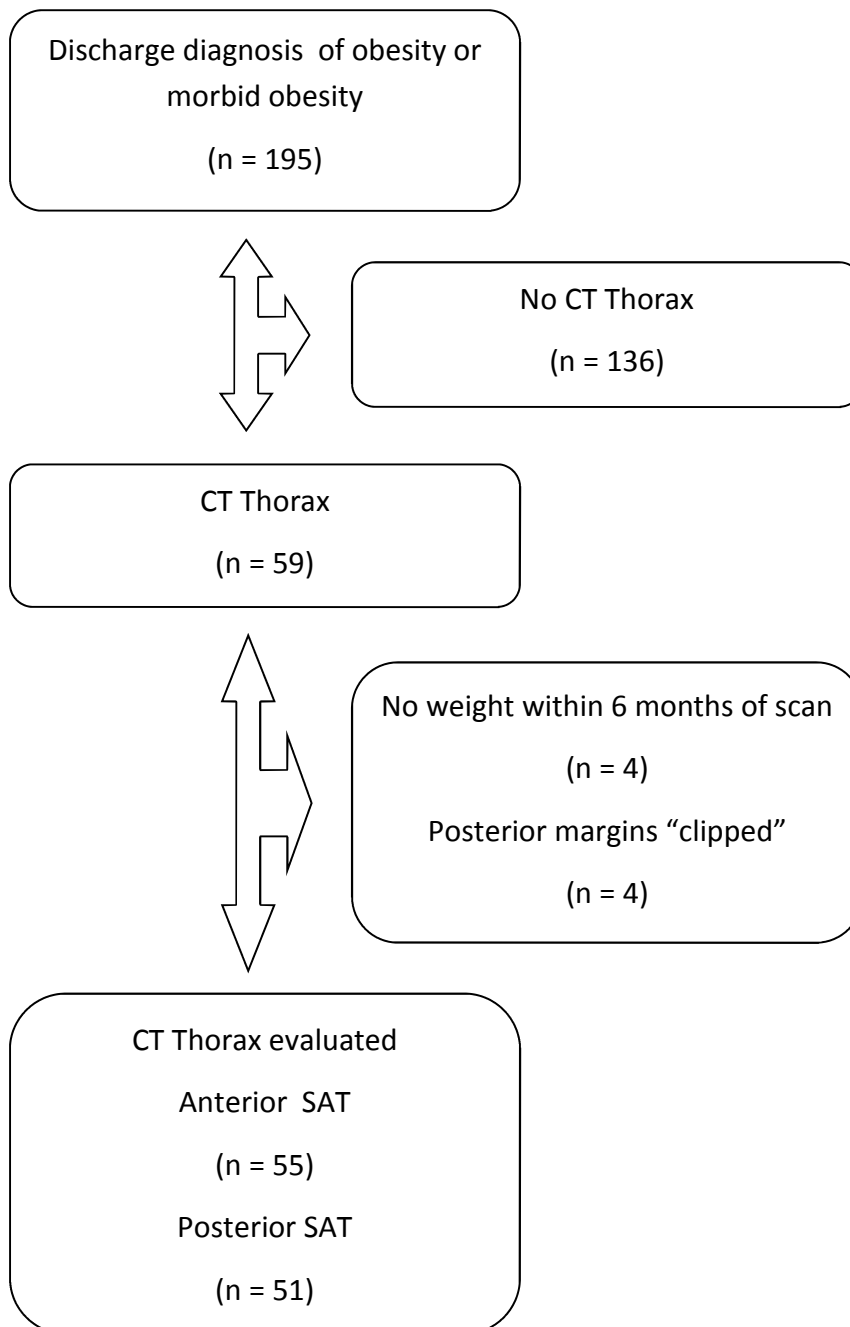

Supplement: Supplementary file 1 — Additional file 1. Flow of patients through study. CONSORT type flow diagram. [file 13104_2017_2918_MOESM1_ESM.pdf]
